# The efficacy of Tai Chi for depression: A protocol for systematic review and meta-analysis

**DOI:** 10.1097/MD.0000000000028330

**Published:** 2022-02-04

**Authors:** Guojian He, Xiucai Zhang, Ting Yan, Jiayi Wang, Qi Li, Tianyu Liu, Youn-Poong Oh

**Affiliations:** a Physical Education Department, Hebei University of Economics and Business, Shijiazhuang, Hebei, China; b School of International Education, University of Electronic Science and Technology, Chengdu, Sichuan, China; c School of Sport, Chengdu University of Traditional Chinese Medicine, Chengdu, Sichuan, China; d Clinical Medical College, Chengdu University of Traditional Chinese Medicine, Chengdu, Sichuan, China; e Department of Physical Education, Kunsan National University, Gunsan, Korea.

**Keywords:** depression, mental disorder, mind-body exercise, Tai Chi

## Abstract

**Background:**

Depression is a commonly occurring and recurrent mental disorder cross the world. Tai Chi is a traditional Chinese mind-body exercise which could be used to treat mental disorders including depression. This study aimed to conduct a systematic review and meta-analysis to assess the efficiency of Tai Chi for patients with depression.

**Methods:**

This protocol follows the Preferred Reporting Items for Systematic Reviews and Meta-Analyses protocol statement. Literature will be searched at PubMed, EMBASE, Web of Science, Cochrane Library, China Biology Medicine Database, China National Knowledge Infrastructure, Technology Journal Database, and Wan Fang database from the start date to September 2021. The Review Manager 5.3 software will be used to manage literature. After literature screening, 2 reviewers will extract data from the respects of general information, methodology, and results. The data analysis will be conducted with Review Manager and Stata 16 software, and the publication bias and literature quality will be both evaluated.

**Results:**

The results will contain the evaluation of clinical efficacy of Tai Chi practice for depression, as well as the assessment of literature quality and publication bias.

**Conclusion:**

The current review will provide new evidence on whether and to what extent patients with depression can benefit from Tai Chi practice.

Registration number: DOI: 10.17605/OSF.IO/AUDNQ (https://osf.io/audnq).

## 1 Introduction

Depression is a commonly occurring and recurrent mental disorder, which mainly manifested as depressed mood, anhedonia, diurnal variation, neurovegetative symptoms and even with suicidal ideation.^[1,2]^ It was reported that more than 350 million people suffered from depression around the world,^[3]^ and it have been the 4th leading cause of disability worldwide.^[4]^ The current therapy of depression is dominated by pharmacotherapy. The tricyclic antidepressants, antipsychotic drugs, and monoamine oxidase inhibitors are the most used drugs in clinical.^[5]^ However, although medications have definite clinical efficacy, their serious side-effects cannot be ignored.^[6,7]^ Therefore, searching for a greener, safer, and more effective complementary and alternative therapy for depression is increasingly becoming a consensus among patients and doctors.^[8,9]^

Tai Chi is a traditional healthcare exercise that originated in ancient China. As a typical mind-body exercise, Tai Chi is not only able to treat musculoskeletal diseases,^[10,11]^ but also has excellent therapeutic effects on neuropsychiatric disorders.^[12,13]^ The recent study demonstrated that 28-weeks of Tai Chi practice could significantly reduce the depression scores and improve the quality of life of older women, and the treatment effects remained 4 weeks after stopping intervention.^[14]^ Similarly, this consistent therapeutic effect of Tai Chi on depressive symptoms has also been proven in a variety of other diseases.^[15–17]^ However, there is no comprehensive overview of these clinical trials about Tai Chi interventions for depression right now. Therefore, we will conduct this systematic review and meta-analysis following the Preferred Reporting Items for Systematic Reviews and Meta-Analyses (PRISMA) statement,^[18]^ to assess the efficiency of Tai Chi for depression, comprehensively.

## 2 Methods

### 2.1 Study registration

This protocol follows the Preferred Reporting Items for Systematic Review and Meta-Analysis Protocols 2015 statement^[19]^ and has been registered at the OSF (https://osf.io/audnq). The registration number is DOI: 10.17605/OSF.IO/AUDNQ.

### 2.2 Inclusion and exclusion criteria

#### 2.2.1 Study design

The randomized controlled trials, nonrandomized controlled trials, and prospective cohort studies will be included, while the retrospective cohort studies, cross-sectional studies, case-control studies, and other nonprospective studies will be excluded. The included literature should be the peer-viewed original article. The case report, meta-analysis, review article, conference proceeding, theses, and other unpublished study will be excluded.

#### 2.2.2 Participants

Studies focusing on patients with depression will be included. The patients should be diagnosed with the criteria of 17-item Hamilton Rating Scale for Depression,^[20]^ the Diagnosis and Statistical Manual of Mental Disorders,^[21]^ the International Classification of Diseases,^[21]^ or the Chinese Classification of Mental Disorders.^[22]^ Studies that depression is regarded as an accompanying symptom rather than a major complaint of patients will be excluded. The age, gender, and ethnicity of the participants will not be restricted.

#### 2.2.3 Interventions

Studies that Tai Chi is regarded as the main intervention will be included. The genre of Tai Chi will not be restricted. Among Chen-style, Yang-style, Wu-style, and Sun-style Tai Chi will be included. Among 24-style, simplified 24-style, 36-style, and 48-style Tai Chi will also be considered.^[23]^

#### 2.2.4 Controls

There are no restrictions on the treatment of control subjects. The control group might be treated with the oral medicine, placebo, psychotherapy, lifestyle modification, and other exercise therapy. It should be noted that if the control group is another type of Tai Chi, the study will be excluded.

#### 2.2.5 Outcomes

The primary outcome of the included study should be the changes of depression score after treatment. the depression score could be measured by the Hamilton Depression Scale,^[24]^ the Self-Rating Depression Scale,^[25]^ and the Beck Depression Inventory.^[26]^

The secondary outcomes are changes of the accompanying symptoms of depression after treatment, which mainly included anxiety, stress, panic, insomnia, fatigue, and so on. Moreover, the depression-related quality of life score, such as SF-36 will also be taking into consider as secondary outcomes.

### 2.3 Search strategy

Electronic searching will be conducted in PubMed, EMBASE, Web of Science, Cochrane Library, China Biology Medicine Database, China National Knowledge Infrastructure, Technology Journal Database, and Wan Fang database. The time span of electronic searching is from the start date to September 2021. The example of searching strategy is showed in Table 1 and will be migrated to the other electronic databases.^[27]^ Thereafter, the snowballing search strategy will be utilized to find more eligible studies according to the reference of enrolled studies. The Review Manager (version5.3) software will be used to manage the searched literature.

**Table 1:** Search strategy in PubMed (English) and CNKI (Chinese).

PubMed	CNKI
#1 Tai Ji [MeSH Terms]	#1 SU=‘Tai Ji’
#2 Tai Ji [Title/Abstract]	#2 SU=‘Tai Ji Quan’
#3 Tai Chi[Title/Abstract]	#3 TI=‘Tai Ji ∗’
#4 Tai Chi Chuan [Title/Abstract]	#4 AB=‘Tai Ji ∗’
#5 Tai Ji Quan [Title/Abstract]	#5 #1 OR #2 OR#3 OR #4
#6 #1 OR #2 OR#3 OR #4 OR #5	#6 SU=’Yi Yu Zheng’
#7 Depression [MeSH Terms]	#7 SU =’Yi Yu’
#8 Depress∗ [Title/Abstract]	#8 TI=‘Yi Yu∗’
#9 Depressive Disorder [MeSH Terms]	#9 AB=‘Yi Yu∗’
#10 Major Depressive Disorder [MeSH Terms]	#10 #6 OR #7 OR#8 OR #9
#11 Major Depressive Disorder [Title/Abstract]	#11 #5 AND #10
#12 #7 OR #8 OR #9 OR #10 OR #11	
#13 #6 AND #12	

CNKI = China National Knowledge Infrastructure.

### 2.4 Study screening and data extraction

**Figure 1:**
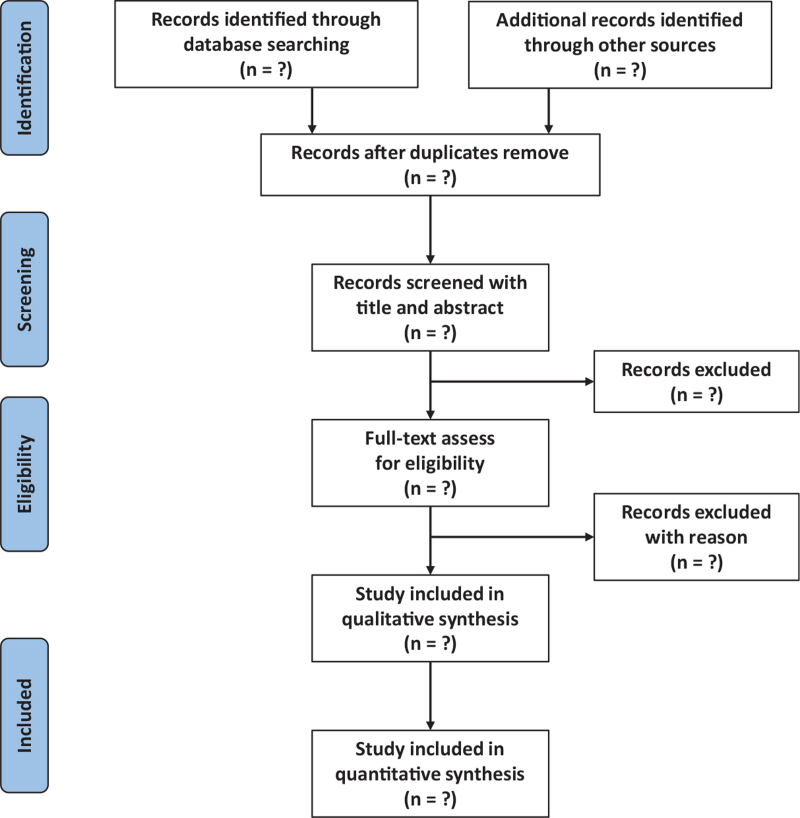
Flow chart of literature selection.

After removing the duplicates, 2 independent reviewers (G H and C G) will screen the literature with 2 steps. Step one is title and abstract review, and step two is full-text review. If there are some disagreements between these 2 reviewers in study screening, a third reviewer (CL) will make the final decision. The flowchart of literature selection is presented in Figure 1.

Data extraction will also be carried out by the same 2 reviewers in parallel. The extracted data includes the general information (first author, year of publication), the details of methodology (participant, sample size, intervention, control, primary outcome, secondary outcome, and study design), and the results (results of primary and secondary outcomes, security, and adverse events). The primary outcome of this review is the changes of depression score, and the secondary outcome contain accompanying symptoms of depression and the depression-related quality of life score.

When data extraction, any missing information or questions about the above data will be solved by contacting the corresponding authors of literature.

### 2.5 Statistical analysis

The Review Manager software will be utilized to create a flow chart of data selection process referring the PRISMA statement.^[18]^ Review Manager and Stata 16 software will be applied to conduct the meta-analysis. The weighted mean differences will be calculated with 95% confidence intervals for continuous variables. All the dichotomous values will be displayed as risk ratio and 95% confidence intervals. The *Q* test and *I*^*2*^ test will be selected to evaluate the heterogeneity of different literature. If the heterogeneity of literature is not significant (*P* ≥ .1 or *I*^*2*^ < 50%), a fixed effects model will be introduced to analyze the pooled effects, while when heterogeneity is statistically significant (*P* < .1 or *I*^*2*^ ≥ 50%), a random effects model will be used for data analysis. The forest plots will be utilized to reflect the corresponding changes in treatment effects.

### 2.6 Assessment of publication bias

If more than 10 literature is enrolled in meta-analysis, the funnel plot will be drawn to reflect the publication bias. A symmetrical funnel plot implies no publication bias.

### 2.7 Assessment of literature quality

Quality of each literature will be assessed by 2 independent reviewers with the Cochrane tool.^[28]^ Risk of each study will be scored as high, low, or unclear.

## 3 Discussion

The systematic review and meta-analysis conducted on the high-quality published clinical trials is regarded as the highest-rated evidence in evidence-based medicine.^[29]^ As early as 2014, a systematic review focusing on the effects of Tai Chi on depression, anxiety, and psychological well-being have been conducted. The results of this review showed that Tai Chi practice might have beneficial effects for various mental disorders including depression, anxiety, and general stress.^[30]^ However, due to the low-level evidence of the included studies, the authors declared that this result need further validation.

In recent years, as the traditional mind-body exercises such as Tai Chi, Yoga and Qigong are increasingly accepted by mainstream medicine,^[31,32]^ studies on Tai Chi practice for depression have grown rapidly.^[32–34]^ Therefore, we propose to conduct this comprehensive and systematic review to assess the clinical efficacy of Tai Chi intervention for depression, with the guidance of PRISMA statement. The results of this review will provide new evidence on whether and to what extent patients with depression can benefit from Tai Chi practice. Furthermore, we hope to propose a clinical superiority program about Tai Chi practice for the management of depression through the literature analysis, so as to enrich the treatment method of depression and improve the clinical efficacy.

## Author contributions

**Conceptualization:** Tianyu Liu, Youn-Poong Oh, Guojian He.

**Data curation:** Xiucai Zhang.

**Formal analysis:** Xiucai Zhang, Ting Yan.

**Investigation:** Xiucai Zhang, Ting Yan, Jiayi Wang.

**Methodology:** Ting Yan, Jiayi Wang, Qi Li.

**Project administration:** Qi Li, Youn-Poong Oh.

**Resources:** Guojian He, Tianyu Liu.

**Software:** Xiucai Zhang.

**Visualization:** Jiayi Wang.

**Writing – original draft:** Xiucai Zhang.

**Writing – review & editing:** Ting Yan, Tianyu Liu, Youn-Poong Oh, Guojian He.
